# DAF-16/FoxO in *Caenorhabditis elegans* and Its Role in Metabolic Remodeling

**DOI:** 10.3390/cells9010109

**Published:** 2020-01-02

**Authors:** Aleksandra Zečić, Bart P. Braeckman

**Affiliations:** Laboratory of Aging Physiology and Molecular Evolution, Department of Biology, Ghent University, 9000 Ghent, Belgium; Aleksandra.Zecic@UGent.be

**Keywords:** DAF-16/FoxO, aging, longevity, metabolic shift, trehalose, glycogen, fat

## Abstract

DAF-16, the only forkhead box transcription factors class O (FoxO) homolog in *Caenorhabditis elegans*, integrates signals from upstream pathways to elicit transcriptional changes in many genes involved in aging, development, stress, metabolism, and immunity. The major regulator of DAF-16 activity is the insulin/insulin-like growth factor 1 (IGF-1) signaling (IIS) pathway, reduction of which leads to lifespan extension in worms, flies, mice, and humans. In *C. elegans daf-2* mutants, reduced IIS leads to a heterochronic activation of a dauer survival program during adulthood. This program includes elevated antioxidant defense and a metabolic shift toward accumulation of carbohydrates (i.e., trehalose and glycogen) and triglycerides, and activation of the glyoxylate shunt, which could allow fat-to-carbohydrate conversion. The longevity of *daf-2* mutants seems to be partially supported by endogenous trehalose, a nonreducing disaccharide that mammals cannot synthesize, which points toward considerable differences in downstream mechanisms by which IIS regulates aging in distinct groups.

## 1. Introduction

Over the past decades, the free-living nematode *Caenorhabditis elegans* has been developed into a prominent model organism in aging research. This is primarily due to its short lifespan, the ease of culturing and experimentation [1], the fully sequenced and well-annotated genome [2,3], and ample availability of forward and reverse genetic screens [4]. Identification of DAF-16, the *C. elegans* homolog of the forkhead box transcription factors class O (FoxO), as one of the key regulators of the aging process [5,6,7] prompted massive attention and a wealth of studies aimed at identifying its upstream regulators, downstream targets and the mechanisms by which it controls aging.

Originally, *daf-16* was identified in studies on the genetic basis of dauer formation [8,9]. The dauer larva is the most specialized diapause stage of *C. elegans* and it is crucial for survival in the spatially and temporally restricted microbe-rich patches of rotting plant material, which this worm naturally inhabits [8,9,10,11]. Under adverse conditions, L1/early L2 larvae develop into a dauer, an alternative L3 stage. Dauer entry is triggered by a continuously secreted dauer (“crowding”) pheromone and this process is further modulated by temperature and food availability [12,13]. Dauer larvae are long-lived and considered non-aging (as the post-dauer lifespan is not affected by the time spent in dauer stage) [14] and highly resistant to various types of stress [15]. When the environment becomes hospitable again, dauers molt into L4 larvae and development is continued as usual.

## 2. Identification of DAF-16: The Genetics of Dauer Arrest

Early studies to identify genes regulating dauer arrest characterized two classes of *daf* (abnormal dauer formation) mutants: dauer constitutive (*daf-c*) and dauer defective (*daf-d*) [8,9]. The *daf-16* mutations were classified as *daf-d* because these mutants failed to form dauers under dauer-inducing conditions [8]. *daf-2* mutants, on the other hand, were *daf-c* since they formed dauers even under favorable conditions [8]. Furthermore, dauer entry in *daf-2* mutants was partially or entirely suppressed by *daf-16* mutations [16]. In the same decade, the first single-gene mutation causing lifespan extension in *C. elegans* was identified: *age-1* (allelic with *daf-23*) [17,18,19,20]. In the early 1990s, mutation in *daf-2* was found to double *C. elegans* lifespan and this effect required functional *daf-16*, thus linking the genetics of dauer formation and longevity [5]. In the years to come, molecular cloning and characterization showed that *daf-2* encodes a receptor tyrosine kinase homolog [21], *age-1* a phosphatidyl inositol 3-kinase (PI3K) homolog [20], and *daf-16* a FoxO transcription factor [6,7]; all acting together in an insulin/insulin-like growth factor 1 (IGF-1) signaling (IIS) pathway.

## 3. DAF-16/FoxO Structure and Isoforms

The forkhead box (Fox) gene family encodes transcription factors characterized by a winged-helix DNA-binding domain (‘forkhead box’) and is evolutionarily conserved in species ranging from yeast to humans [22,23]. These transcription factors have regulatory roles in a plethora of cellular processes, such as differentiation, apoptosis, DNA repair, cell cycle arrest, metabolism, stress and immune response, and longevity [24,25,26]. In mammals, there are 19 subclasses of Fox transcription factors (A to S) based on sequence homology [23] and the FoxO subclass contains four members encoded by distinctive genes: FoxO1 (FKHR), FoxO3 (FKHRL1), FoxO4 (AFX), and FoxO6 [23,27]. In invertebrates, only one member of the FoxO subfamily has been characterized: DAF-16 in *C. elegans* [6,7] and dFOXO in *Drosophila melanogaster* [28].

In *C. elegans,* five different DAF-16 isoforms are transcribed from three distinctive promoters of a single genetic locus: *daf-16a1*/*a2*, *daf-16b*, and *daf-16d/f* [29,30,31,32]. These isoforms differ in their tissue distribution and function as indicated by multiple studies using transcriptional and translational reporters, promoter swapping, mutants, and RNA interference (RNAi) [29,30,31,33,34]. While *daf-16a1*/*a2* and *daf-16d/f* are expressed in almost all tissues [29,30,31], *daf-16b* expression is primarily enriched in the pharynx, somatic gonad, and neurons [29,31,33], and this distribution is driven by divergence at the isoforms’ N-termini [31,34]. Furthermore, *daf-16a1*/*a2* and *daf-16d/f* have dominant roles in controlling dauer arrest and lifespan [29,31,34], while *daf-16b* is required for pharynx remodeling during dauer formation [29,31] and neurite outgrowth during development [33].

To regulate the expression of its targets, DAF-16 binds to a consensus DNA motif TTGTTTAC, known as the DAF-16 binding element (DBE) [35]. Analysis of the 1 kb region upstream of the putative DAF-16 targets revealed that the DBE is overrepresented in promoter regions of both Class I (upregulated in *daf-2* mutants) and Class II (downregulated in *daf-2* mutants) genes, suggesting that DAF-16 also functions as a transcriptional repressor [36]. However, by using DamID, Schuster et al. [37] demonstrated that DAF-16 acts solely as a transcriptional activator of Class I genes through binding to DBE. A microarray study by Murphy et al. [36] identified a second overrepresented motif: the GATA-like sequence CTTATCA, later named DAF-16 associated element (DAE) [38]. Rather than DAF-16, DAE is bound by PQM-1, a transcriptional factor with a key role in controlling the expression of Class II genes and their downregulation in *daf-2* mutants, thus accompanying DAF-16’s role in IIS-mediated longevity [39].

## 4. The IIS Pathway: A Central Regulator of DAF-16 Activity

The insulin/IGF-1 signaling pathway is an evolutionarily conserved phosphorylation cascade that controls growth, metabolism, and reproduction in response to nutrient availability in all eukaryotes. Moreover, this pathway has a well-established role in regulating aging as reduced IIS leads to lifespan extension in *C. elegans* [5,18], *D. melanogaster* [40,41], and *Mus musculus* [42,43].

The IIS cascade is activated by binding of insulin-like peptides (ILPs) to the membrane receptor tyrosine kinase. The *C. elegans* genome encodes 40 putative ILPs that are largely expressed in neurons and interneurons [44,45,46]. However, the differential roles of these peptides are not yet fully explored. These ILPs are not generally redundant, rather, they have distinct functions and regulate each other transcriptionally within a complex signaling network that affects *C. elegans* development, stress resistance, and longevity [47]. A recent study functionally characterized all 40 ILPs in vivo, by creating transgenic lines overexpressing individual peptides pan-neuronally and scoring multiple phenotypes [48]. The study unraveled functions of 35 ILPs and classified them as agonists, antagonists, or pleiotropic to IIS. Moreover, eight ILPs were shown to have specific functions (e.g., INS-15, INS-21, and INS-22 are IIS antagonists with roles in dauer formation, fat accumulation, and lifespan of L1 arrested larvae, respectively) [48]. *D. melanogaster* has eight ILPs (DILPs) encoded by distinct genes [49,50], while in mammals the ILP superfamily contains 10 members of which only insulin, IGF-1, and IGF-2 are related to *C. elegans* ILPs and bind to tyrosine kinase receptors [51,52,53,54,55]. In striking contrast to the number of ILPs, there is only one insulin receptor (IR) in *C. elegans*—DAF-2. In humans, on the other hand, there are several tyrosine kinase receptors (IR, IGF-IR, IGF-IIR, and a hybrid insulin receptor-related receptor—IRR) that bind insulin, IGF-1, and IGF-2 with different affinities to elicit diverse and complex physiological effects [56].

Upon insulin-like ligand binding, the *C. elegans* DAF-2 receptor auto-phosphorylates, which results in the recruitment and activation of the p110 catalytic subunit of PI3K/AGE-1 [20]. AGE-1 catalyzes conversion of phosphatidylinositol 4,5-bisphosphate (PIP_2_) into phosphatidylinositol 3,4,5-trisphosphate (PIP_3_) [57]. In humans, the p110 catalytic subunit is recruited to the membrane by PI3K adaptor subunits that bind to the phosphotyrosines of the insulin receptor substrate (IRS) scaffold proteins [58]. The *C. elegans* genome encodes the IRS and PI3K adapter unit homologs, *ist-1* and *aap-1*, respectively [59]. However, *aap-1* and *ist-1* are deemed dispensable for AGE-1/p110 catalytic subunit activation by DAF-2, at least in a wild-type background [59]. Elevated levels of PIP_3_ result in activation of the 3-phosphoinositide-dependent kinase-1 homolog PDK-1 [60], which, in turn, phosphorylates and activates the Akt/PKB homologs AKT-1 and AKT-2 [61] and the serum- and glucocorticoid-inducible kinase SGK-1 [62]. In mammals, both AKT and SGK can inhibit FoxO3A by phosphorylation but differ in preference for regulatory sites [63,64]. In *C. elegans*, the regulatory role of SGK-1 is less clear. While SGK-1 phosphorylates DAF-16 in vitro [62], it does not influence DAF-16 subcellular localization in vivo, but probably exerts its regulatory function by affecting other proteins that interact with DAF-16 [65]. Phosphorylation of DAF-16 by AKT-1 and AKT-2 promotes its sequestration in cytoplasm through the association with 14-3-3 scaffold proteins PAR-5 and FTT-2 [66,67]. Under the conditions of reduced IIS due to stress or mutations in *daf-2* or any of the pathway kinases, DAF-16 translocates into the nucleus where it modulates transcription of its targets [36,38,68,69].

## 5. IIS-Independent Regulation of DAF-16 Activity

To fulfill its diverse roles, DAF-16 integrates signals from multiple upstream pathways that act in parallel to IIS. c-Jun N-terminal kinase JNK-1, a member of the mitogen-activated protein kinase (MAPK) family, and CST-1, a Ste20-like kinase 1 (MST1) homolog, directly phosphorylate DAF-16, leading to its nuclear translocation and activation in response to heat and oxidative stress [70,71]. Furthermore, overexpression of both *jnk-1* and *cst-1* results in a *daf-16*-dependent lifespan extension [70,71]. Another kinase that activates DAF-16 via direct phosphorylation is the AMP-activated protein kinase (AMPK) [72]. AMPK was shown to phosphorylate DAF-16 in vitro at least at six different residues, but it does not increase its nuclear translocation [72,73]. In turn, DAF-16 is a direct transcriptional activator of *aakg-4*, which encodes the regulatory γ subunit of AMPK, thus suggesting the existence of a positive feedback loop that further activates DAF-16 and the expression of its target genes [73]. This notion is additionally supported by the upregulation of *aakg-4* in *daf-2* mutants and lifespan shortening of *daf-2* due to *aakg-4* RNAi or mutation [73]. The nutrient-sensing target of rapamycin (TOR) signaling pathway also converges on DAF-16. More specifically, genetic inhibition of TORC1 leads to increased transcription of *daf-16* and the nuclear translocation of a single DAF-16 isoform, DAF-16d/f, resulting in lifespan extension [74]. In addition to DAF-16, this longevity phenotype also requires activation of SKN-1 [74]. Under the conditions of intermittent fasting, however, TORC1 promotes DAF-16 nuclear translocation and activation of its target genes in a LET-363/TOR- and RHEB-1-dependent manner [75]. Finally, removal of germline cells results in nuclear translocation and activation of DAF-16 in the intestine and this is facilitated by *kri-1*, which codes for an intestinal ankyrin-repeat protein [76], and the microRNA *mir-71* [77].

## 6. Identification of DAF-16 Targets

In order to uncover underlying molecular mechanisms of *C. elegans* IIS mutant longevity, several approaches have been used with the aim to first identify DAF-16 transcriptional targets (reviewed in [78]). Bioinformatic approach by Lee et al. [79] identified 17 orthologs in *C. elegans* and *D. melanogaster* that contained the consensus DBE within 1 kb of their promoters. Subsequent functional analysis of these candidates in *C. elegans* by RNAi revealed that they regulate longevity, dauer formation, and fat storage [79]. Likewise, initial transcriptomic studies based on expression microarrays and serial analysis of gene expression (SAGE) identified DAF-16-dependent upregulation of genes involved in metabolism, cellular stress, and antimicrobial response [36,38,80,81]. A subset of these genes was functionally assayed for lifespan phenotypes by RNAi, and despite the fact that the vast majority had a significant effect, not a single treatment completely abolished the longevity of *daf-2* mutants like *daf-16* RNAi, pointing toward a complex network of multiple effector genes to regulate aging [36]. In subsequent studies, chromatin immunoprecipitation (chIP) [82,83,84] and DamID [37] were performed to distinguish between direct and indirect targets of DAF-16. Oh et al. identified 88 putative targets that had at least one DAF-16 binding site in their promoter region using chIP on non-synchronized cultures of *daf-2* worms [82]. A DamID approach detected 65 ‘high-confidence’ targets, i.e., genes that are targets of DAF-16 and are regulated by IIS, which were also enriched for Class I genes identified in the previous microarray study [37]. Many of the differentially expressed DAF-16 targets identified in *daf-2* mutants by genome-wide studies are shared with the transcriptome of dauer larvae [38,68,85]. These observations are supported by multiple proteomic [86,87,88,89,90] and metabolomic [91,92] studies, which also highlighted the extensive remodeling of metabolism in *daf-2* mutants, reminiscent of hypometabolic dauers. This is not surprising given that dauers are also long-lived and supports the idea that *daf-2* mutants could potentially rely on the heterochronically activated dauer survival program for their longevity assurance. In line with this is the discovery that the TGF-β signaling pathway regulates both longevity and dauer formation and this occurs partially through interactions with IIS and changes in DAF-16 cellular localization [93]. Further evidence that supports the notion that *daf-2* are “adult dauers” is that, at 20 °C, adults of the Class 2 *daf-2(e1370)* mutant show mild dauer-like characteristics, such as body darkening, gonad shrinkage, and early-life decline in spontaneous movement [94,95,96]. On the contrary, Ewald et al. have demonstrated that the lifespan extension due to reduced IIS can be independent of the dauer program [95]. This is the case when *daf-2(e1370)* are grown at 15 °C or the *daf-2* RNAi is performed at 15, 20, or 25 °C, all of which are the conditions where dauer-like traits are absent and require SKN-1 for lifespan extension [95]. Thus, the requirement of the heterochronically activated dauer program for *daf-2* longevity appears to be context-dependent. In the following sections, we will discuss aspects of dauer physiology manifested in *daf-2* mutants and their importance for *daf-2* longevity.

## 7. DAF-16-Mediated Enhanced Stress Resistance in Long-Lived *daf-2* Mutants

In *C. elegans*, reduced IIS confers extension of lifespan as well as elevated resistance to various types of stress, including heat [97], oxidative [98,99], UV [100], osmotic [101], hypoxic [102,103], and heavy metal [104] stress. Likewise, long-lived IIS mutants also show increased resistance to bacterial pathogens [105], reduced bacterial colonization, and enhanced clearance of pathogenic bacteria [106]. This wide range of stress responses is mediated by joint activity of DAF-16 with transcription factors such as HSF-1 [107,108], SKN-1 [109,110,111], SMK-1 [112] and, with regards to innate immunity, the p38 MAPK pathway [113]. Could increase in stress resistance be the key mechanism of IIS mutant longevity given the correlation between these two phenotypes? Concordant with this idea, numerous studies have demonstrated upregulation of superoxide dismutases (SODs), catalases, and glutathione S-transferases in long-lived IIS mutants [36,80,98,114,115]. However, both double deletion of *sod-2* and *sod-3* (mitochondrial) [116] and *sod-1* and *sod-5* (cytosolic) [117] render IIS mutants sensitive to oxidative stress but do not abolish their longevity. Deletion of *sod-4* (extracellular) even further extends *daf-2* lifespan [117]. Moreover, a quintuple mutant with a complete loss of SOD activity has a normal lifespan despite being increasingly sensitive not only to oxidative but also osmotic, heat, and cold stress [118]. Finally, a recent study has shown a similar effect in *daf-2* worms: complete lack of SOD activity fully suppresses resistance to oxidative and heat stress but has a negligible effect on longevity [119]. Altogether these results imply that stress resistance and longevity can be experimentally uncoupled and that elevated activity of antioxidant enzymes is dispensable for *daf-2* longevity. If not supporting longevity, what could possibly be the role of increased antioxidant defense? Taking into consideration *C. elegans* ecology, high antioxidant activity in dauers is crucial for the survival of anhydrobiosis and rehydration, which are accompanied by high levels of reactive oxygen species [120]. In *daf-2* mutants that do not naturally undergo these cycles of anhydrobiotic survival, elevated antioxidant levels could potentially be only an integral part of the heterochronically activated dauer program that is unrelated to lifespan extension.

## 8. The Metabolism in *daf-2* Mutants is Extensively Remodeled

**Figure 1:**
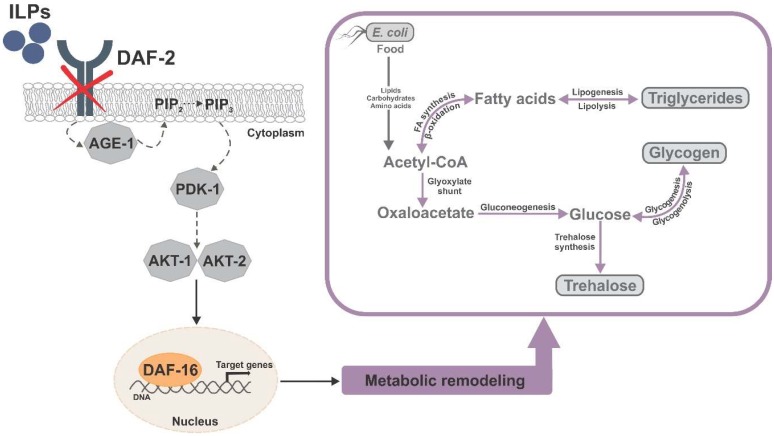
Simplified graphic summary of the DAF-16-dependent metabolic remodeling in the *Caenorhabditis elegans daf-2* mutant, with emphasis on the major carbon stores and their interconversion.

In addition to elevated stress resistance, another aspect of dauer physiology is reiterated in *daf-2* mutants: a massive restructuring of intermediary metabolism [86,89,91] (Figure 1). In dauers, nonessential and energy-costly metabolic functions, such as feeding, growth, and reproduction, are suppressed [121], and all the internal energy stores are used for long-term structural maintenance and stress resistance [122]. Prior to dauer formation, young larvae accumulate triglycerides as intestinal and hypodermal lipid droplets [123], an energy reserve that can be consumed in a slow and strictly controlled manner to ensure survival of extended periods without food [123,124]. Additionally, dauers accumulate glycogen, which could serve as a readily available short-term energy source for locomotion and nictation, especially in the first weeks after dauer formation when they are highly motile [122].

Similarly, *daf-2* mutants have large lipid [7,21,89,125] and glycogen stores [89,126] in their intestine and hypodermis, as shown by different histological, biochemical, and ultrastructural studies. Transmission electron microscopy (TEM) images also indicate higher amounts of glycogen in *daf-2* body-wall muscles [89]. Furthermore, *daf-2* mutants show increase in de novo fatty acid synthesis, as demonstrated by a ^13^C isotope-labeling approach, and this effect is entirely dependent on DAF-16 activity [127]. This finding appears to be in contrast with upregulation of many genes involved in β-oxidation in *daf-2* worms at protein level [89]. Namely, fatty acid β-oxidation is inhibited by high concentrations of malonyl-CoA coming from increased fatty acid synthesis. These discrepancies could be explained by the different stages of worms used in the isotope-labeling and proteomic studies, i.e., late L4 larval stage and day 2 adults, respectively [89,127]. Indeed, age-dependent changes in lipid content in *daf-2* mutants point toward a metabolic switch from lipogenesis during development and early adulthood (up to day 2) to tightly controlled lipolysis throughout the remaining life [89], reminiscent of non-feeding dauer larvae [123,124]. Concordant with this notion is also the reduced level of the fatty acid synthase homolog FASN-1 in adult *daf-2* worms [89]. However, it is crucial to highlight that the described increase in lipid synthesis and storage refers to mutants in a canonical allele *daf-2(e1370)* [7,89,125,127] and that certain alternative lifespan-extending alleles such as *e1368* and *m577* show no such phenotype [127], suggesting that the bulk increase in lipid content per se is not directly correlated with longevity in IIS *daf-2* mutants. On the other hand, altering lipid composition toward increase in unsaturated fatty acids could potentially play a role. Consistent with this, IIS longevity mutants show DAF-16-dependent upregulation of fatty acid desaturase FAT-7 [36] and elevated levels of monounsaturated fatty acids (MUFAs) [92,128,129] as well as MUFA-rich triglycerides [129].

Glycogen is essential for survival of acute hyperosmotic stress in *C. elegans* as a readily available reservoir for the production of glycerol in a pathway mediated by AMPK [130]. Its protective role was also demonstrated in *daf-2* mutants in conditions of osmotic stress and anoxia [126,131]. Lifespan effects of glycogen in worms fed on standard and glucose-rich diet were evaluated by knocking down genes involved in glycogen synthesis and breakdown, i.e., glycogen synthase (*gsy-1*) and glycogen phosphorylase (*pyg-1*) [132]. *gsy-1* knockdown depletes glycogen reserves, however, it causes only a negligible effect on *daf-2* longevity on both diets [132]. *pyg-1* RNAi results in a further increase in glycogen content in *daf-2* worms (relative to controls without a knockdown), however, this treatment drastically shortens lifespan only on a high glucose diet, suggesting that accumulating excess glucose in form of glycogen in these conditions accelerates aging [132]. These observations were recently confirmed in a study that has also demonstrated that reduction of *gsy-1* leads to a metabolic shift toward an increase in stored trehalose, which is crucial for alleviating detrimental effects of high dietary glucose in both wild type and *daf-2* worms [133]. Moreover, this beneficent effect of trehalose requires the functional DAF-16d/f isoform [133]. The role of glycogen in *daf-2* physiology is not entirely clear. It appears that these worms live even slightly longer when glycogen is depleted by *gsy-1* knockdown [133] (in contrast with [132]), yet, GSY-1 is one of the most upregulated proteins in *daf-2* mutants, in agreement with the large glycogen accumulations observed in these worms [89]. It is plausible that accumulation of this sugar is a result of the activated dauer program with the sole purpose of protection against stress.

Another sugar that could possibly be the main player in *daf-2* longevity is the nonreducing disaccharide trehalose. It is a major circulating sugar in insects, where it is used as an energy source for fueling glycolysis [134] and its protective role against desiccation [135,136], heat [137], and osmotic [101,138] stress is shown in diverse organisms. In *C. elegans* and the anhydrobiotic nematode *Aphelenchus avenae*, there are two trehalose phosphate synthase genes, *tps-1* and *tps-2*, responsible for synthesis of trehalose [139,140]. These genes are the downstream targets of *daf-2/daf-16* signaling and are highly upregulated in dauers and *daf-2* mutants [36,80,89], in accordance with increased trehalose levels in these worms [91,101,141]. In dauer larvae, trehalose is essential for survival of extreme desiccation [135]. Furthermore, this effect requires a fully active glyoxylate cycle that allows utilization of fat as a major carbon source for trehalose synthesis [142]. *C. elegans* has a single protein with two enzymatic functions (isocitrate lyase and malate synthase) that is responsible for the glyoxylate shunt reactions [143] and exists in two isoforms: ICL-1 (GEI-7) and the poorly characterized C08F11.14 [126,142]. ICL-1 is expressed in mitochondria and it catalyzes conversion of isocitrate and acetyl-CoA into glyoxylate and succinate and subsequent conversion of glyoxylate into malate. While succinate can further fuel the tricarboxylic acid (TCA) cycle, malate can be converted into oxaloacetate and used for the gluconeogenesis to generate trehalose [142,144]. Certainly, impairment of ICL-1 function results in a severe impediment of dauer desiccation tolerance due to a reduced amount of trehalose in these worms [142]. Likewise, RNAi knockdown of *icl-1* shortens lifespan in the *daf-2* mutants *mu150* and *e1370*, by up to ~23% and ~27%, respectively, probably by a similar mechanism [145]. These observations are supported by heavy upregulation of *icl-1* in dauers [68,142,146] and *daf-2* mutants [36,89,145] and a recent metabolic model that predicts greater production of trehalose from fatty acids by activated glyoxylate cycle in microaerobic conditions [147]. Finally, simultaneous RNAi knockdown of *tps-1* and *tps-2* shortens lifespan in *daf-2* mutants, also indicating that the endogenous trehalose is at least partially responsible for *daf-2* longevity [141].

The protective role of trehalose is ascribed to the preservation of membrane organization through maintaining native packaging of lipids, prevention of protein denaturation, and aggregation and clearance of formed aggregates through induction of autophagy [89,120,133,135,148,149]. Moreover, trehalose was shown to jointly act with a group of intrinsically disordered proteins (late embryogenesis abundant, LEA) to exert its protective function both in vitro and in vivo [148]. There is only one LEA homolog in the *C. elegans* genome, encoded by *lea-1*, and it is essential for dauer desiccation tolerance [120]. Not surprisingly, *lea-1* is shown to be upregulated in *daf-2* worms, yet again pointing toward the heterochronic activation of the dauer survival program in these mutants [86,89]. Considering the importance of trehalose for longevity and its role in alleviating detrimental effects of glucose-rich diet in *C. elegans* [133,141,145], it would be interesting to explore the tissue dependency of these effects and the mechanisms of trehalose transport between different tissues.

The described restructuring of intermediary metabolism in long-lived *daf-2* mutants is an evolutionarily conserved strategy of small organisms with restricted mobility, such as fungi [150], some plants [151], and invertebrates [135,152], to survive desiccation. This tolerance relies heavily on mechanisms that are absent in vertebrates (e.g., trehalose synthesis, glyoxylate shunt activation, LEA proteins) and that, at least partially, contribute to *daf-2* longevity. Even though the FoxO transcription factors have been linked to human longevity, the underlying mechanisms by which this is achieved could differ substantially between worms and humans due to differences in the details of their physiology, organismal complexity, and environmental challenges these organisms face in nature [153,154].

## 9. Conclusions

The *C. elegans* FoxO homolog DAF-16 is the main output of IIS with a central role in regulating aging, which is an evolutionarily conserved function. However, DAF-16-mediated changes in *daf-2* mutants are reminiscent of the hypometabolic dauer larvae and the longevity of these worms seems to rely, at least in part, on mechanisms that are not present in humans, such as synthesis of trehalose and activation of the glyoxylate cycle. Furthermore, it appears that certain aspects of *daf-2* physiology are a consequence of a heterochronically activated dauer program with a role in stress resistance, but not longevity. On the other hand, the discovery of DAF-16 in *C. elegans* and its conserved role in aging across metazoans stemmed from studies on the genetics of dauer arrest. In addition to dauer formation, IIS also regulates many other aspects of *C. elegans* physiology (e.g., metabolism, autophagy, proteostasis), which have been linked to aging and diseases. Moreover, IIS-mediated longevity in *daf-2* mutants can also be achieved independently of a genetic dauer program, in a SKN-1-dependent manner [95]. Finally, certain single nucleotide polymorphisms in the DAF-16 ortholog, FoxO3A, have been associated with human longevity [155]. Taken together, these findings suggest that uncovering more details of IIS-mediated longevity in *C. elegans* could provide potentially valuable targets for treatment of aging and age-related diseases in humans.
